# An Optical Test Strip for the Detection of Benzoic Acid in Food

**DOI:** 10.3390/s110807302

**Published:** 2011-07-25

**Authors:** Hairul Hisham Hamzah, Nor Azah Yusof, Abu Bakar Salleh, Fatimah Abu Bakar

**Affiliations:** 1 Faculty of Science, Universiti Putra Malaysia, 43400 Serdang, Selangor, Malaysia; 2 Faculty of Applied Science, Universiti Teknologi Mara, 26400 Jengka, Pahang, Malaysia; E-Mail: hhisham@pahang.uitm.edu.my; 3 Institute of Technology and Advance Material (ITMA), Universiti Putra Malaysia, 43400 Serdang, Selangor, Malaysia; 4 Faculty of Biotechnology and Biomolecular Sciences, Universiti Putra Malaysia, 43400 Serdang, Selangor, Malaysia; E-Mail: abubakar@biotech.upm.edu.my; 5 Faculty of Food Science and Technology, Universiti Putra Malaysia, 43400 Serdang, Selangor, Malaysia; E-Mail: fatim@putra.upm.edu.my

**Keywords:** optical biosensor, test strip, tyrosinase, benzoic acid

## Abstract

Fabrication of a test strip for detection of benzoic acid was successfully implemented by immobilizing tyrosinase, phenol and 3-methyl-2-benzothiazolinone hydrazone (MBTH) onto filter paper using polystyrene as polymeric support. The sensing scheme was based on the decreasing intensity of the maroon colour of the test strip when introduced into benzoic acid solution. The test strip was characterized using optical fiber reflectance and has maximum reflectance at 375 nm. It has shown a highly reproducible measurement of benzoic acid with a calculated RSD of 0.47% (n = 10). The detection was optimized at pH 7. A linear response of the biosensor was obtained in 100 to 700 ppm of benzoic acid with a detection limit (LOD) of 73.6 ppm. At 1:1 ratio of benzoic acid to interfering substances, the main interfering substance is boric acid. The kinetic analyses show that, the inhibition of benzoic is competitive inhibitor and the inhibition constant (K_i_) is 52.9 ppm. The activity of immobilized tyrosinase, phenol, and MBTH in the test strip was fairly sustained during 20 days when stored at 3 °C. The developed test strip was used for detection of benzoic acid in food samples and was observed to have comparable results to the HPLC method, hence the developed test strip can be used as an alternative to HPLC in detecting benzoic acid in food products.

## 1. Introduction

Benzoic acid is a white solid that is slightly soluble in water and is an extensively used preservative. It is generally effective in controlling mould and inhibiting yeast growth and also in preventing a wide range of bacterial attacks. Although this preservative prevents or delays nutritional losses due to microbiological, enzymatic, or chemical changes of foods during its shelf life, it is harmful at higher than permitted safety levels [1]. The maximum permitted concentrations of benzoic acid in each type of food are controlled by legislation. And the concentration of naturally occurring benzoic acid in several foods should not exceed an average value of 1,000 mg/kg of food [2]. Maximum concentrations reported for benzoic acid added to food for preservation purposes were in the range of 2,000 mg/kg of food [3]. Therefore, the analytical determination of benzoic acid is not only important for quality assurance purposes but also for consumer interest and protection.

Cases of urticaria, asthma, rhinitis, or anaphylactic shock have been reported following oral, dermal, or inhalation exposure to benzoic acid. The symptoms appear shortly even at low doses and disappear within a few hours. Information regarding skin reactions caused by benzoic acid in the general population is limited [4].

Various traditional methods have been reported for the analysis of benzoic acid in foodstuffs, such as thin layer chromatography (TLC) [5], high performance liquid chromatography (HPLC) [6,7] enzyme-linked immunosorbent assay (ELISA) and other immunochemical techniques. However, these methods are expensive, slow, require well trained operators and in some cases, require steps of extraction or sample pre-treatment which increases the time of analysis [1].

Biosensor technology is based on a specific recognition element in combination with a transducer for signal processing. Since their inception, biosensors have been expected to play significant analytical roles in medicine, agriculture, food safety, homeland security, environmental and industrial monitoring [8]. Biosensor devices incorporate a biological sensing element and an electrical transducer to produce an electrochemical, optical, or mass signal in proportion to the concentration of analyte in a sample. Among these transducers, the optical biosensor offers simplicity and sensitivity in the development of a detection system. There are two main areas in optical biosensors, which determine changes in light absorption between the reactants and products of a reaction or measure the light output by a luminescent process. The optical biosensor system has many advantages compared to the other sensor system due to the capability of remote and multiple sensing [9]. Besides that, optical biosensors are not subject to interference from electric fields and are easy to miniaturize, which can lead to the development of very small, light, and flexible sensors [10].

Polyphenol oxidase (PPO), also called tyrosinase, is a binuclear copper monooxygenase enzyme containing metalloid-protein. It is widespread in Nature throughout the polygenetic scale from bacteria to mammals and takes part in a large number of biological reactions. Tyrosinase catalyzes the *ortho*-hyroxylation of monophenols to form *ortho*-diphenols (cresolate activity) and the oxidation of *ortho*-phenols to *ortho*-quinones (catchecolase activity), or both, at the expense of oxygen as an electron-acceptor [1]. Tyrosinase has a binding site with an affinity for aromatic compounds (the substrate site) and another with an affinity for metal binding (oxygen site). Tyrosinase has various inhibitors that affect the enzymatic reaction and benzoic acid is one of the inhibitors of tyrosinase [11].

The use of an amperometric biosensor to detect benzoic acid in food products based on the inhibition mechanism of PPO using a novel phenol biosensor has been reported [1,4,12–14]. However the use of an optical biosensor to detect benzoic acid based on the inhibition mechanism of tyrosinase using a novel phenol biosensor has not yet been reported. In this paper we describe the use of a phenol biosensor for the development of an optical biosensor for benzoic acid based on immobilized tyrosinase, phenol, and MBTH into filter paper using polystyrene as a polymeric support. The sensing scheme is based on the decreasing intensity of a maroon colored product associated with the reaction of *o*-quinone and 3-methyl-2-benzothiazolinone hydrozone (MBTH). The effect of pH, volume of tyrosinase, interference substances and kinetic study of the inhibition are described in detail.

## 2. Experimental Section

### 2.1. Apparatus

Reflectance was measured using an optical fiber reflectance spectrophotometer and the instrumental parameters were controlled by the operating software of a SpectraSuite spectrometer (model SD2000, Ocean Optics Inc.). All data were collected and processed by this software.

### 2.2. Preparation of Reagents

Tyrosinase (98% purity) from mushroom (EC 1.14.18.1, 4.741 units mg^−1^) and phenol (99% purity) was purchased from Sigma. MBTH (99% purity) was purchased from Merck. Polystyrene (mw ∼250K) was purchased from Merck. Tetrahdyrofuran (99% purity) was purchased from Sigma. Tyrosinase (10 mg) was dissolved in 0.05 M phosphate buffer (pH 6.5, 100 mL). The tyrosinase solution was filtered with a filter paper and stored at 3 °C. MBTH powder (0.54 g) was dissolved in deionized water (250 mL). The stock solution of MBTH was stored in a refrigerator maintained at 3 °C. Phenol crystals (0.38 g) were dissolved in deionized water (500 mL). A stock solution of 1,000 ppm of benzoic acid was prepared by dissolving 1.00 g of benzoic acid in 1,000 mL of deionized water. A series of standard solutions were prepared by appropriate dilution of the stock solution.

### 2.3. Preparation of Food Samples (Soy Sauce and Oyster Sauce)

Soy sauce and oyster sauce (5 mL, purchased from a local supermarket) was accurately pipetted into a 50 mL volumetric flask and diluted up to the mark with deionized water. The pH was adjusted to pH 6.5 with 0.5 M of sodium phosphate dibasic. The sample was filtered to remove suspended matter. Then the whole sample was diluted with 100 mL deionized water. A 5 mL aliquot of this solution was diluted with 100 mL deionized water.

### 2.4. Preparation of Test Strip

In this fabrication of test strip, an impregnating method was used. It is one of the possible enzyme immobilization procedures. This method involves impregnating a matrix carrier (filter paper) with an enzymatic testing solution includes tyrosinase, phenol, and chromogen (MBTH). Filter paper (Whatman, No. 4) was purchased from Sigma. The filter papers were cut into pieces of 1.0 × 1.5 cm. A mixture of tyrosinase (2 mL), 0.01 M phenol (6 mL), and 0.01 M MBTH (3 mL) was left for 30 min until a complete change of color (colorless to dark maroon) occurred. The filter paper was dipped into the mixture solution and dried at room temperature. The test strip was then impregnated with 1 g of polystyrene in 30 mL tetrahydrofuran (THF) and again dried at room temperature. The test strip was stored at 3 °C.

### 2.5. Measurement Procedure

The prepared test strips were introduced into 5 mL of benzoic acid solution (100 ppm) for 30 min. The change in color was measured using an optical fiber reflectance spectrophotometer. Reflectance was recorded between 300 to 600 nm of wavelength.

## 3. Results and Discussion

### 3.1. Spectral Study

**Figure 1.**
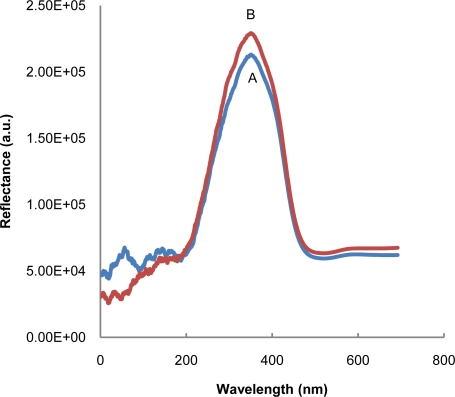
Reflectance spectra for test strips (A) before and (B) after being introduced into 100 ppm of benzoic acid solution.

Figure 1 shows the reflectance spectra before (A) and after (B) introducing the test trip into100 ppm of benzoic acid solution.

The inhibition effect of benzoic acid on tyrosinase enzyme causes an increase in reflectance due to the decreasing intensity of the maroon color on the test strip. The reactions involved are shown below:

(1) Phenol + Tyrosinase → Dihydroxybenzene

(2) $$\text{Dihydroxybenzene}\;+\;\text{Tyrosinase}\;\to \;o-\text{quinone}\;+\;H_{2}O$$

(3) o−quinone + MBTH (colorless) → o−quinone−MBTH (maroon)

In these enzymatic reactions, phenol is a substrate for tyrosinase, benzoic acid is the inhibitor and MBTH is the color indicator. According to previous works, benzoic acid is a reversible inhibitor (and it can be proved in this study by analyzing the kinetic investigation between tyrosinase, phenol and benzoic acid, see Section 3.6). Based on the theory, the reversible inhibitor is not only bound to the enzyme but also to the enzyme-substrate complex, the active center is usually deformed and its function is thus impaired [15]. When this happen, the amount of enzyme-substrate complex will be decreased, due to the inhibition effect of benzoic acid on normal function of tyrosinase. Therefore, the quantities of benzoic acid can be measured based on the decreased amount of enzyme-substrate complex.

### 3.2. pH Optimization

**Figure 2.**
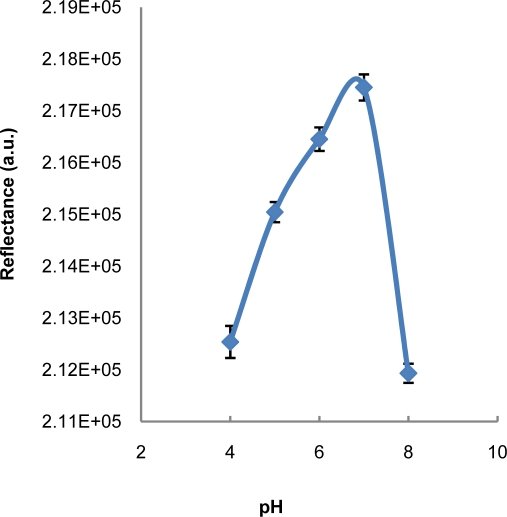
The effect of pH on the detection of benzoic acid (100 ppm) using the developed test strip.

The pH of an enzyme solution can affect the overall catalytic activity because enzymes have a native tertiary structure that is sensitive to pH and the denaturation of enzymes can occur at extreme pH values [16]. Therefore, the influence of pH on the detection of benzoic acid was carried out in this study in order to identify the optimum pH. Figure 2 shows the result of the pH study and the optimum pH for the detection of benzoic acid is identified at pH 7. Therefore, pH 7 was selected for further analytical procedure. Previous works reported that the optimum pH for the inhibitory action of benzoic acid on the enzymatic activity of tyrosinase was obtained at a pH range of 6–7 [1,4,12,13]. The study on pH is limited to pH 8 only due to the fact that the enzyme will denature at pH higher than this value.

### 3.3. Reproducibility of the Test Strip

Reproducibility study of the developed test strip based on ten replicates (n = 10) revealed an encouraging result. Reproducibility refers to the successive runs made by using the method developed to evaluate discrepancies in its responses. The RSD for reproducibility of the developed method was calculated to be 0.47%. Small RSD values calculated for this method indicate a good precision of the method developed.

### 3.4. Effect of Amount of Tyrosinase on the Detection of Benzoic Acid

**Figure 3.**
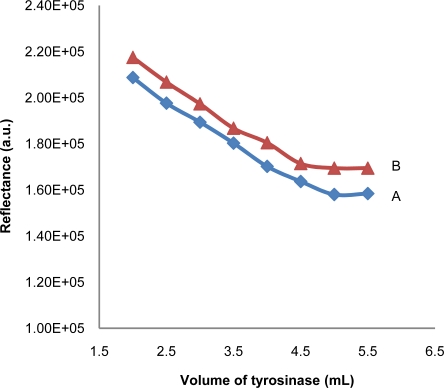
The effect of amount of tyrosinase immobilized into the developed test strip (A) before and (B) after being introduced into100 ppm of benzoic acid solution.

The effect of amount of tyrosinase immobilized on the test strip was also studied and the result is shown in Figure 3. The intensity of reflectance decreases with the increase in tyrosinase volume until a constant value is reached for both with and without benzoic acid. Increase in tyrosinase volume leads to the increase of the enzyme-substrate complex formed [17] and the intensity of the maroon color of the test strips. This plateau region is reached when all available tyrosinase has been converted to enzyme-substrate complex. Based on this observation, volume of 3.5 mL was chosen for the fabrication of further test strips.

### 3.5. Analytical Application of the Test Strip

**Figure 4.**
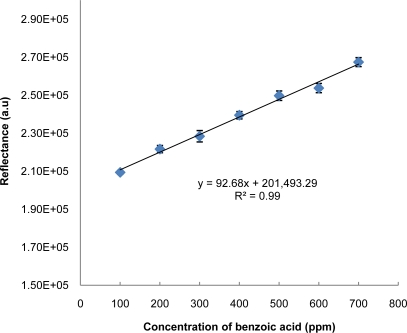
The calibration plot for detection of benzoic acid using the developed test strip.

A linear response was observed when the detection was carried out for benzoic acid concentration in the range of 100–700 ppm (Figure 4). The limit of detection (LOD) of the method defined as the concentration equivalent to a signal of blank plus three times the standard deviation of the blank is calculated to be 73.6 ppm. Previous studies on the inhibitive detection of benzoic acid based on the polyaniline-polyacrylonitrile composite matrix [1] and inhibition of benzoic acid on the polyaniline-polyphenol oxidase biosensor [14] yielded a linear dynamic range of 2–100 ppm with LOD of 0.024 ppm and 4–100 ppm with LOD of 0.037 ppm. In our study, the LOD is higher than the previous works. However, the LOD obtained is still under permitted levels (<1,000 ppm) according to the Malaysian Food Act 1983 [2]. The applicability of the optical test strip in simple and rapid monitoring of benzoic acid in food compared to electrochemical based biosensor is important. The developed test strip has proven to be reliable, sensitive enough, cost effective and requires a simple analytical procedure for the detection of benzoic acid compared with an electrochemical based biosensor. To our knowledge, very little research has been carried out on optical based biosensors for the detection of benzoic acid. In addition, the optical-based biosensor is a convenient method, because this method offers simple and cheaper detection procedures compared to the usage of electrode (electrochemical biosensor). Other advantages of using the optical biosensor method including no requirement of a reference electrode, no interference from electroactive species, the electrode fouling problem is avoided and there is no complicated sample pretreatment.

### 3.6. Kinetic Study and Mechanism of the Inhibitory

**Figure 5.**
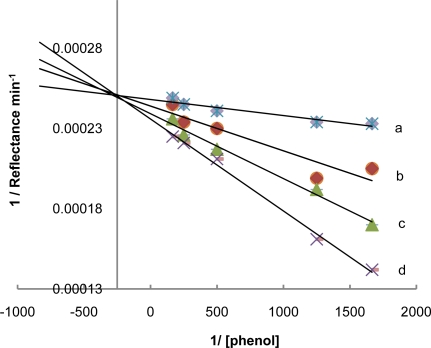
The Lineweaver-Burk plots for phenol in the absence (a) and in the presence of 150 ppm (b), 250 ppm (c), and 350 ppm (d) benzoic acid.

The inhibition mechanism can be studied by examining the relationship between the response of the sensor to the substrate and the inhibitor concentration [18]. In this study, the inhibition kinetics of tyrosinase by benzoic acid was investigated. The relationship of enzyme activity in the absence and presence of different concentrations of benzoic acid was interpreted using the double-reciprocal Lineweaver-Burk plots. Figure 5 shows the plots of 1/reflectance min^−1^ *versus* 1/[phenol] that gives a straight lines with different slopes intersecting one another in the Y axis. Higher concentration of benzoic acid resulted in an increase in the slope value, indicating that inhibition of benzoic acid on the enzyme is a reversible reaction.

**Figure 6.**
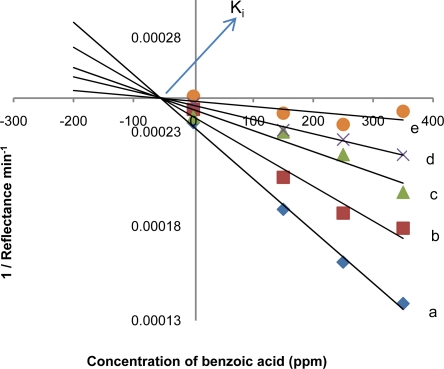
The Dixon plots of benzoic acid inhibition in the presence of 0.002 M (a), 0.004 M (b), 0.006 M (c), 0.008 M (d), and 0.01 M (e) phenol.

K_M_ is defined as the concentration of the specific substrate at which a given enzyme yields one-half its maximum velocity [19]. The values of R_max_ remains the same irrespective of the present or absent of the inhibitor while the value of Michaelis-Menten constant (K_M_) increases (Table 1) with increase in inhibitor concentration. From this, it can be concluded that benzoic acid is a competitive inhibitor of the tyrosinase enzyme [1,12,14]. Competitive inhibitor which is structurally related to the substrate may be bound to the enzyme active site and compete with the substrate [1]. From the Dixon plots (Figure 6) the inhibition constant (K_i_) for benzoic acid binding to tyrosinase is 52.9 ppm.

### 3.7. Effect of Possible Interfering Substances

In order to demonstrate the selectivity of the biosensor, a study on the effect of potential interferences in the detection of benzoic acid was carried out. Under optimized experimental conditions, a study on the influence of sodium chloride, citric acid, ascorbic acid, and boric acid on the determination of benzoic acid was carried out and the results are listed in Table 2. The results show that all the interfering substances give positive interferences. From the results listed, boric acid gives the highest interference in the determination of benzoic acid using the developed test strip and led us to conclude that boric acid slightly inhibits tyrosinase and confirms the evidence proved by the previous study reported by Kerry and Earl [20].

### 3.8. Stability of the Test Strip

**Figure 7.**
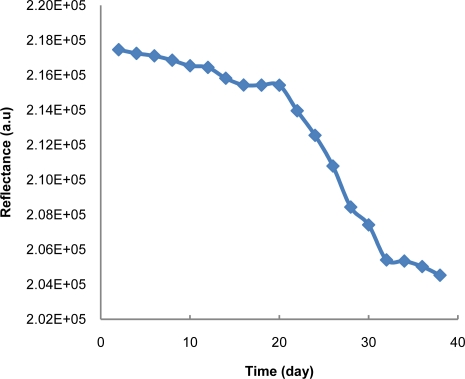
The stability of the developed test strip.

As most of the biological materials, enzyme activity decreases with time [21]. Therefore, sustainability of enzyme activity is important in the development of enzyme-based biosensor. Figure 7 shows the stability of the developed test strip against time. It can be seen from the result that enzyme activity decreases with time. Loss of activity can be accounted to irreversible enzyme denaturation with time and working storage conditions. Theoretically, enzyme activity decreases with time in an almost linear manner [21,22]. The test strips were stored in a freezer at 3 °C during the experiment. The reflectance intensity was sustained up to 20 days and slowly decreased with time in linear manner and it was considered to be non-functional after that [21,22].

### 3.9. Application on Real Sample Analysis

The developed test strip was applied for the detection of benzoic acid in food samples. Soy and oyster sauce (from a local supermarket) were assayed in order to demonstrate the practical usage of the biosensor. The contents of benzoic acid in the samples were calculated using the calibration curve and are listed in Table 3. The results based on the proposed method were compared with the results assayed using the HPLC method. The relative errors were within acceptable limits. The calculated results show that the results determined by the proposed method were in satisfactory agreement with those given by the HPLC method, indicating that it is feasible to apply the developed test strip for the determination of benzoic acid in real samples.

## 4. Conclusions

In previous work, many biosensors based on the inhibition effect of tyrosinase for detecting benzoic acid have been reported [1,12–14]. All the biosensors reported were based on amperometric biosensors, but an optical biosensor based the inhibition effect of tyrosinase for detecting benzoic acid has not yet been reported. In this work, an optical biosensor has been developed for the determination of benzoic acid based on tyrosinase immobilized onto filter paper using polystyrene as a polymeric support. The optimized pH for the detection is at pH 7. The response range of the developed detection method was in the range of 100–700 ppm of benzoic acid. A good reproducibility (RSD = 0.47%) was obtained using the developed method, indicating a reliable detection system. The LOD value was 73.6 ppm. The kinetic interpretation of the optical response of phenol for the tyrosinase based test strip recorded in the absence and in the presence of benzoic acid revealed a competitive process. The inhibition constant (K_i_) is 52.9 ppm. The proposed method is simple, easy to operate, reliable, and able to detect benzoic acid at the permitted level, making it a good alternative to traditional methods for benzoic acid analysis.

## Figures and Tables

**Table 1.** The kinetic constant (K_M_) in various concentrations of benzoic acid.

**[Benzoic Acid] (ppm)**	**K_M_ (M)**	**R_max_**
0	0.000040	4030.96
150	0.000123	4090.87
250	0.000167	4180.60
350	0.000322	4030.96

**Table 2.** The effect of possible interfering substances.

**Substance**	**% Interference**
Sodium chloride	4.7
Citric acid	6.8
Ascorbic acid	13.1
Boric acid	21.2

**Table 3.** Determination of benzoic acid in sauce samples by the developed test strip and HPLC method.

**Sample**	**HPLC method (ppm)**	**Test strip (ppm)**	**Relative errors (%)**
Soy sauce	592.3 ± 0.8	604.0 ± 10	+1.9
Oyster sauce	662.0 ± 16	680.0 ± 5	+2.6

## References

[1] Shan D, Fang Q, Zhue D, Xue H (2007). Inhibitive detection of benzoic acid using a novel phenol biosensor based on polyaniline-polyacrylonitrile composite matrix. Talanta.

[2] Legal Research Board (2006). Text Book of Malaysian Food Act 1983 (Act 281) and Regulations 1996.

[3] (2006). International Programme on Chemical Safety (IPCS): Benzoic Acid and Sodium Benzoate.

[4] Mustafa K, Tuge G, Erhan D (2005). Detection of benzoic acid by an amperometric inhibitor biosensor based on mushroom tissue homogenate. Food Technol. Biotechnol.

[5] Dong C, Meri Y, Chan L (2006). Simultaneous determination of sorbic acid and benzoic acid in food dressing by headspace solid-phase microextractionandgaschromatography. J. Chromatogr. A.

[6] Thomassin M, Cavalli E, Guillaume Y, Guinchard C (1997). Comparison of quantitative high performance liquid chromatography of parabens. J. Pharm. Biomed. Anal.

[7] Valeria A, Lozano Jose MC, Maria SB (2007). Simultaneous determination of benzoic acid in commercial juices using the PLS-2 multivariate calibration methodand validation byhigh performance liquid chromatography. Talanta.

[8] John HTL, Keith BM, Jeremy DG (2008). Biosensor technology, technology push *versus* market pull. Biotech. Adv.

[9] Trettnak W, Reininger FZ, Wolfbeis E (1993). Fiber optic remote detection of pesticides and related inhibitors of the enzyme acetycholinesterase. Sens. Actuat. B.

[10] Klaimer SM, Thomas JR, Franels JC (1993). Fiber optic chemical sensors offer a realistic solution to environmental monitoring needs. Sens. Actuat. B.

[11] Katrin S, Helvi K, Alexender M (1998). Application of a sensitive catechol detector for determination of tyrosinase inhibitors. Anal. Chim. Acta.

[12] Shan D, Li Q, Xue H, Cosnier S (2008). A highly reversible and sensitive tyrosinase inhibition-based amperometric biosensor for benzoic acid monitoring. Sens. Actuat. B.

[13] Morales MD, Morante S, Escarpa A, Gonzalez MZ, Reviejo AJ (2002). Design of Composite amperometric enzyme electrode for the control of the benzoic acid content in food. Talanta.

[14] Li S, Tan Y, Wang P, Kan J (2010). Inhibition of benzoic acid on the polyaniline oxidase biosensor. Sens. Actuat. B.

[15] Aziz A, Hasna M, Ilhame B, Glussppe P (2006). Enzyme inhibition-based biosensors for food safety and environmental monitoring. Biosens. Bioelectron.

[16] Jeong WC, Young KK, In HL, Jun HM, Wong HL (2001). Optical organophosphorus biosensor consisting of acetycholinesterase/vilogen Hetero Langmuir-Blodgett film. Biosens. Bioelectron.

[17] Jaafar A, Musa A, Nadarajah K, Lee YH, Hamidah S (2006). Immobilization of tyrosinase in chitosan film for an optical detection of phenol. Sens. Actuat. B.

[18] Sarmiza ES, Ionel CP (2004). Amperometric study of the inhibitory effect carboxyclic acid on tyrosinase. J. Mol. Catal. B.

[19] Jaafar A, Musa A, Nadarajah K, Lee YH, Hamidah S (2006). Chitosan-based tyrosinase optical phenol biosensor employing hybrid nafion/sol gel silicate for MBTH immobilization. Talanta.

[20] Kerry TY, Earl RN (1957). Mechanism of borate inhibition of diphenol oxidation by tyrosinase. J. Biol. Chem.

[21] Jeong WC, Young KK, Sun YS, In HL (2003). Optical biosensor consisting of glutathione-S-transferase for detection of captan. Biosens. Bioelectron.

[22] Andreou VG, Yannis DC (2002). Novel fiber-optic biosensor based on immobilization glutathione S-transferase and sol-gel entrappedbromcresol green for the determination of atrazine. Anal. Chim. Acta.

